# Juvenile Ossifying Fibroma and Socioeconomic Barriers to Specialty Care: A Pediatric Case Study

**DOI:** 10.7759/cureus.40059

**Published:** 2023-06-06

**Authors:** Lizeth A Acosta, Sarah Aamer, Monica Becher, Jose Cucalon Calderon

**Affiliations:** 1 Pediatrics and Child Health, University of Nevada Reno School of Medicine, Reno, USA; 2 Pediatrics, University of Nevada Reno School of Medicine, Reno, USA

**Keywords:** multidisciplinary approach, telemedicine, socioeconomic disparities, socioeconomic, paediatric otolaryngology, delayed diagnosis, medicaid population, juvenile trabecular ossifying fibroma

## Abstract

Juvenile ossifying fibroma (JOF) is a rare benign neoplastic fibro-osseous tumor commonly found in the maxilla and mandible of children usually between the ages of five and 15. Patients often present with aggressive, painless growth which is well demarcated from surrounding bone resulting in severe facial asymmetry. JOFs have high recurrence rates if not completely resected and should therefore be treated by a multidisciplinary team of physicians including a neurosurgeon to assess cranial nerve function. This case describes a child who presented to the ED after being referred by his primary care provider for facial swelling. The patient was diagnosed with JOF and had a delay in care due to a lack of access to multidisciplinary specialties to provide care due to payer difficulties which placed the patient at high risk of complications.

## Introduction

Juvenile ossifying fibroma (JOF) is a rare, slow-growing tumor seen in children under 15 years of age [1]. This condition has two histological classifications according to the World Health Organization: psammomatous and trabecular type [2]. Trabecular tumors are mostly seen in the mandible while psammomatoid tumors are found near paranasal sinuses, anterior skull base, and orbits [1]. The highly aggressive growth pattern and recurrence rate make it essential to reach an early diagnosis and quickly decide on the most favorable treatment option. Complications of untreated JOF may include headaches, visual symptoms ranging from visual disturbances to progressive blindness, airway obstruction, and progressive craniofacial deformities [3]. Radiological findings in CT and MRI may reveal the mass and lead to a diagnosis. The primary treatment option to consider is gross-total resection [4].

In complex patient cases such as in this report, it is important to assess barriers to health care that may affect patient outcomes. Previous studies comparing public and private insurance beneficiaries consistently reveal more barriers that result in longer wait times and more emergency department visits in public insurance recipients. One study suggests that Medicaid beneficiaries had twice the likelihood to have at least one emergency department visit in 12 months (39.6%) versus private insurance holders (17.7%) [5]. A combination of patient-, provider-, and system-level determinants are perceived to contribute to limited healthcare utilization in this population [6]. The purpose of this case report is to examine the effect of healthcare disparities in the setting of an emergent pediatric presentation of a rare pathology.

## Case presentation

A 14-year-old male was brought to his primary care pediatrician with concerns about worsening facial swelling and epistaxis. This patient, who is part of a single-parent household, was brought in by his father who initially associated the facial swelling with a fall and collision with a table that had occurred a year prior. Upon physical examination, occlusion of the left nostril with an erythematous mass associated with the swollen and protuberant left eye was noted. Father and patient were advised to seek medical attention at the emergency department for MRI and CT imaging to identify the cause of facial swelling. 

**Figure 1:**
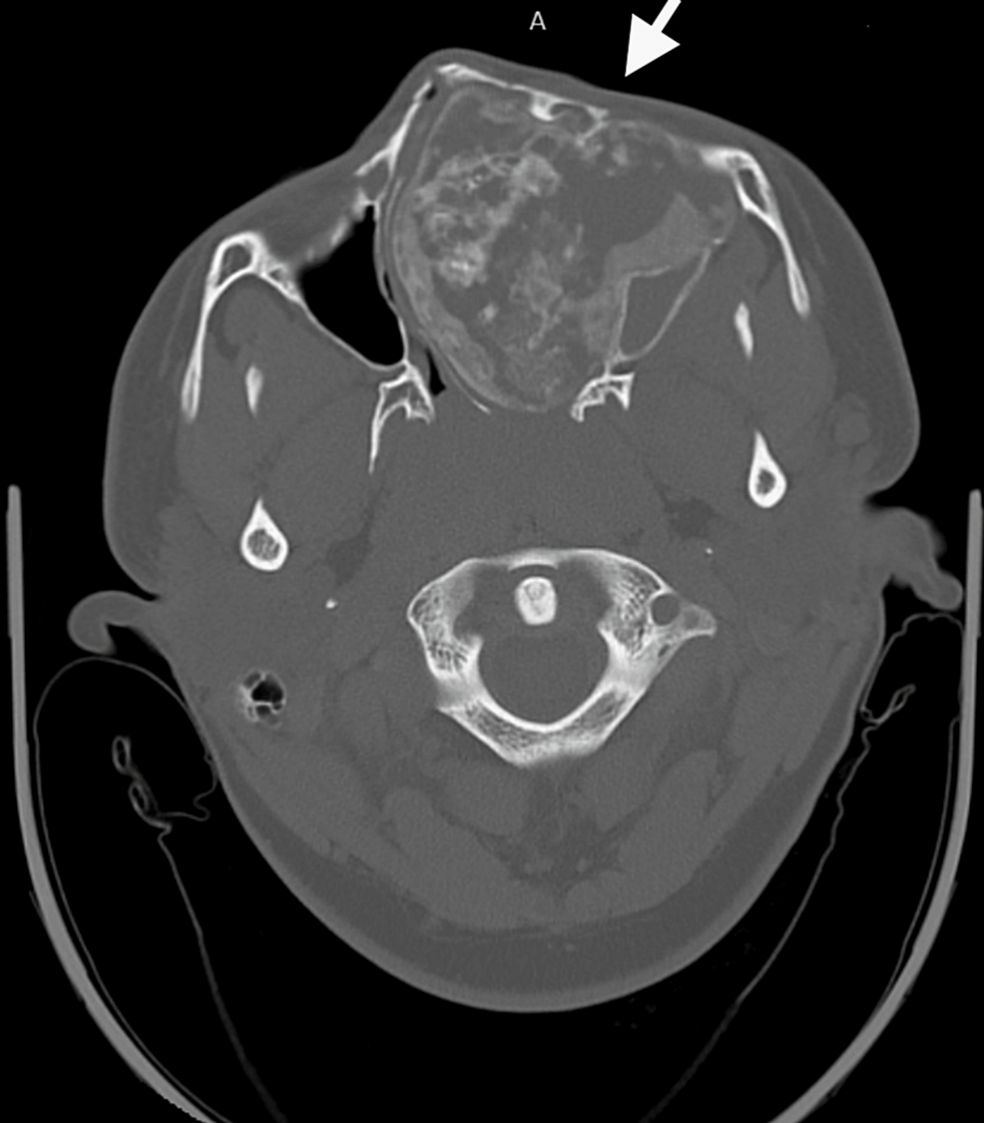
CT head without contrast. Arrow demonstrates large heterogeneous sclerotic and lytic mass involving the left sphenoid, ethmoid, and maxillary sinuses with bony erosion and distortion of the face.

**Figure 2:**
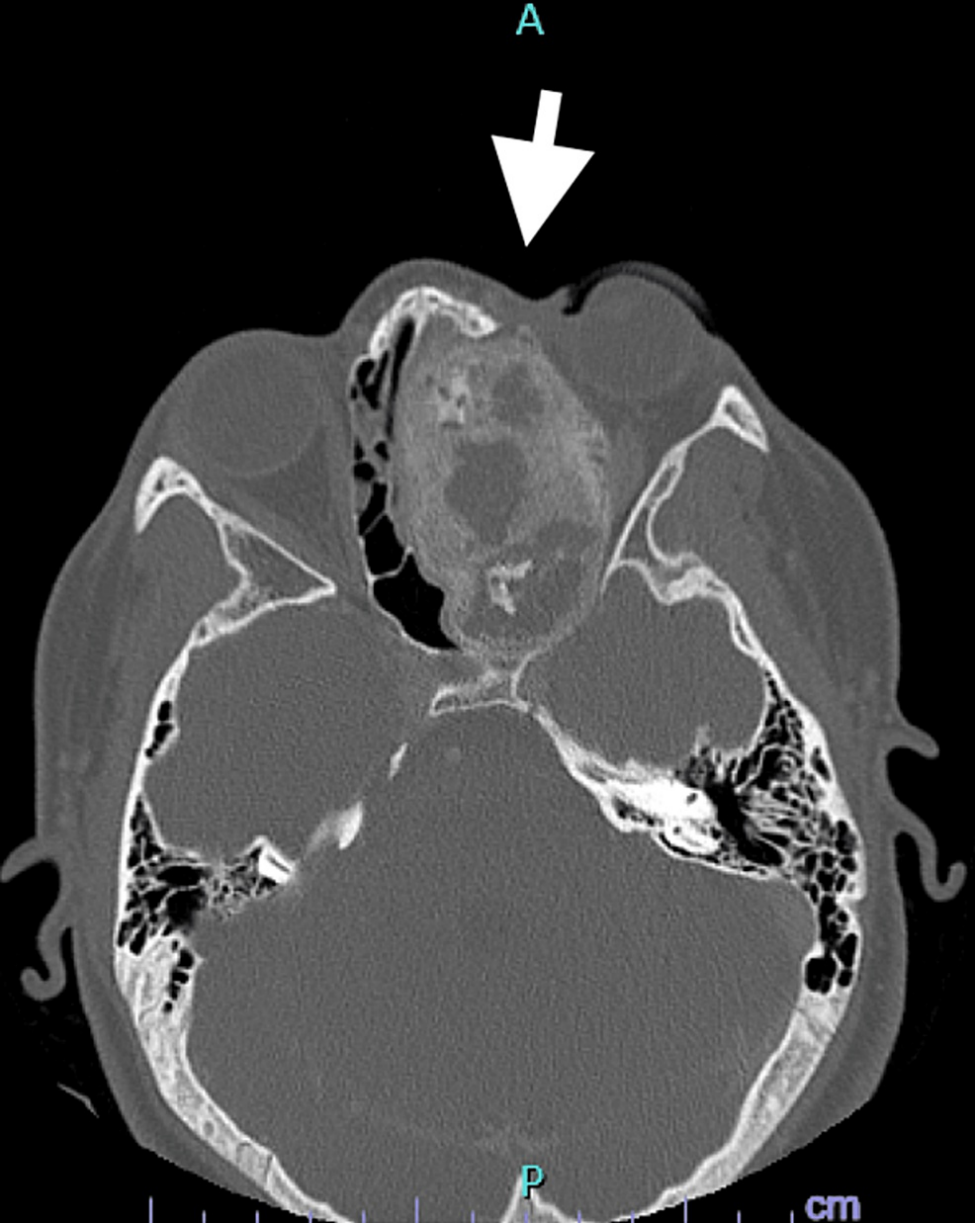
CT-maxillofacial. Large left facial mass with cystic and calcified sclerotic components resulting in severe deformation of face with left proptosis.

**Figure 3:**
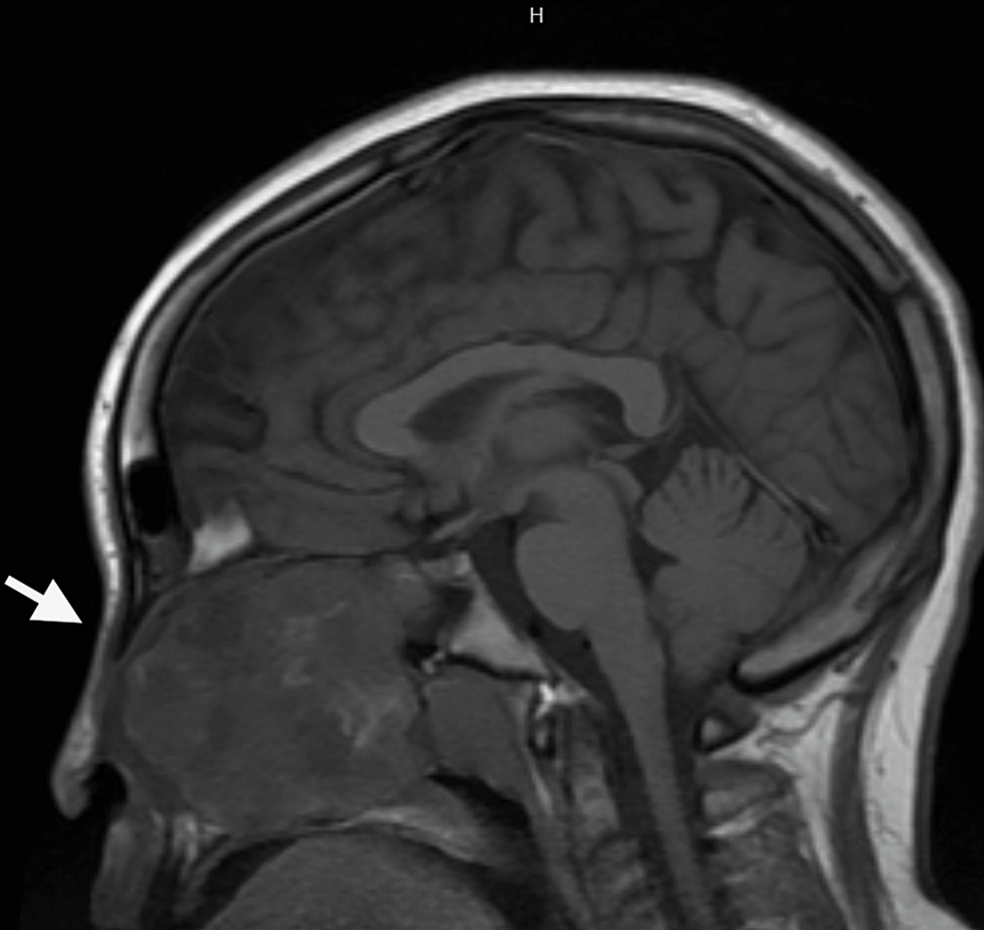
MR-brain sagittal view.

The patient presented to the emergency department the next day and was admitted to the hospital. Blood results indicated microcytic anemia (Hb 11.9 g/dL) and normal white cell count. A CT scan of the head demonstrated a large heterogeneous sclerotic and lytic mass involving the left sphenoid, ethmoid, and maxillary sinuses with bony erosion and distortion of the face (Figure 1). A more focused Maxillofacial CT revealed a large left facial mass with cystic and calcified sclerotic components resulting in severe deformation of the face causing left proptosis (Figure 2). The radiology report favored a neoplastic disease. An MRI of the brain was performed and reported the tumor to likely be a benign ossifying fibroma and recommended a biopsy. Lastly, an MRI of the orbit, face, and neck demonstrated a large well demarcated multicystic enhancing lesion involving the left nasal cavity, left maxillary ethmoid and sphenoid sinus, and left side of the nasopharynx as likely representing a benign ossifying fibroma (Figure 3).

While admitted at the local children’s hospital, ENT and Neurosurgery were consulted and they recommended referral to a larger center out of state with a care agreement reached between both teams. Insurance did not authorize a hospital-to-hospital transfer of care and requested patient be discharged to outpatient follow-up. Insurance approved another consult in another in-state hospital system. 

After repeated attempts by PCP for prompt evaluation by in network subspecialty despite progressively worsening symptoms, the patient was evaluated by ENT and Plastic Surgery at the other in-state hospital system a month later. They agreed with recommendation for specialized care out of state. Insurance requested new referrals and patient was evaluated and treated in a larger, out of state system. Multidisciplinary assessment and care were done by NS and ENT to debulk the base of the tumor via transnasal and sublabial access endoscopically. Findings from rigid nasal endoscopy were reported as: fibro-osseous lesion protruding outside the nasal cavity on the left, and complete obstruction of the nasal cavity from tumor mass effect on the right. The patient was discharged home 13 days after the surgery.

**Figure 4:**
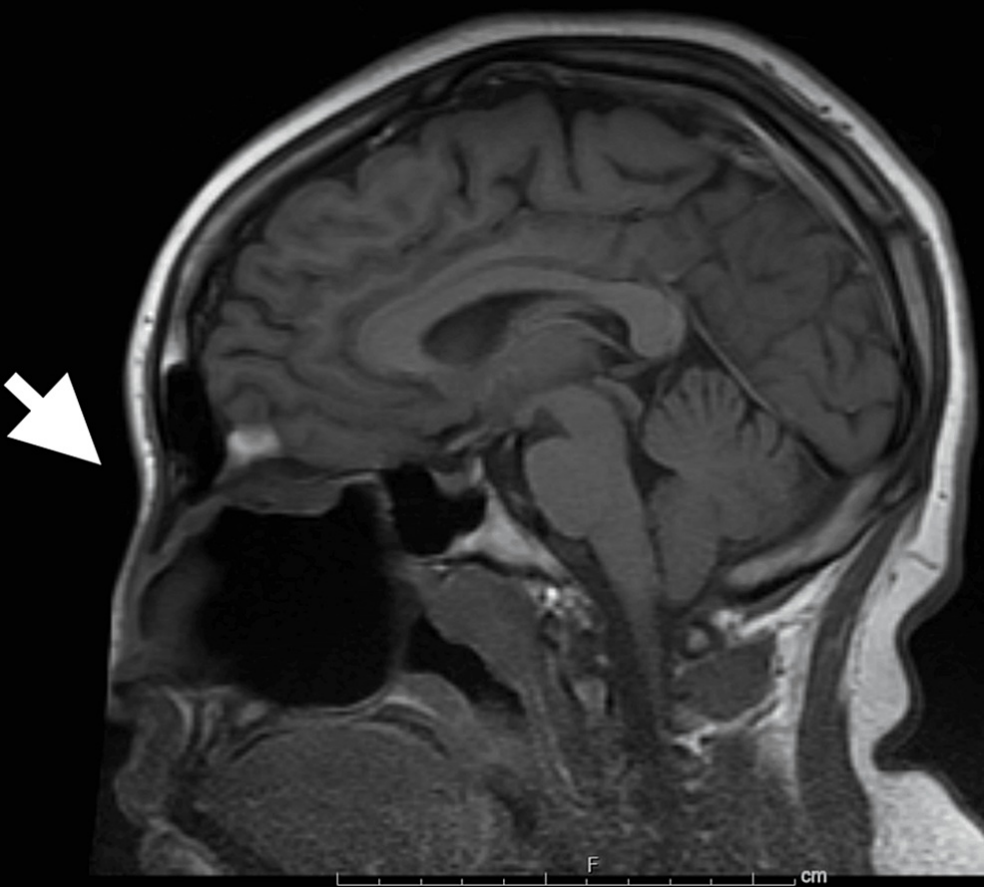
MR-brain. Post-surgical resection of large sinonasal mass.

During the follow-up visit, due to persistent swelling in surgical sites and vision changes, the patient had an updated MRI with and without contrast that showed findings consistent with wound healing and granulation tissue (Figure 4). Vision changes are still being evaluated at the time of this writing. The patient continues to be followed for his care by the same team out of state and by his PCP for his regular medical care.

## Discussion

This case highlights a patient with JOF who experienced a significant delay in surgical intervention despite presenting acutely. While JOF is a benign disease, its characteristic rapid expansion can damage surrounding structures, especially when located in the orbit, an area rich in delicate nerves and blood vessels that can be disrupted by unchecked tumor growth [7]. Because of this, rapid diagnosis and treatment are essential to ensuring a positive outcome in children with this disease process. Typical complicating factors that have been elaborated in previous case studies are related to the difficulty of diagnosis; the relative rarity of JOF makes it challenging for providers to recognize, and histological similarity to cemento-ossifying fibroma can complicate biopsy interpretation [8]. Misdiagnosis can lead to unnecessary chemotherapy and delay of appropriate treatment [9].

This case demonstrates that delayed treatment can result from social and administrative factors even when the diagnosis is rapid and appropriate and highlights the importance of clinician awareness of the barriers patients face in accessing complex specialist care. Common locations for the development of JOF include the maxilla and orbit [1]; surgical resection in these areas requires the involvement of multiple specialists, in this case, an ENT, neurosurgeon, ophthalmologist, and plastic surgeon. Access to complex interdisciplinary procedures is limited by proximity to an appropriate treatment facility, requiring a considerable investment of time and financial resources into travel, including room and board, on the part of the patient and the patient’s family. These burdens were exacerbated by the need of making two separate trips, one for an in-state second opinion after the correct assessment by the initial care team experts recommended care out of state due to the complexity of the case and location of the mass. This delay of a month could have led to critical worsening and long-term health consequences given the fast-progressing nature of the condition and the pending nerve damage to the patient’s eye due to compression by the mass. Of note, this patient has public insurance under Medicaid.

Barriers to in-person visits such as cost and travel time that this patient faced may have been reduced with video telemedicine. Telemedicine consultations have been reported to decrease waiting list time and improve the coordination of specialist services [10]. The expansion of telemedicine intervention in the field of otolaryngology has demonstrated that adequate images allow for remote diagnosis. The effectiveness of remote diagnosis is heavily dependent on the ability to obtain quality images; however, the development of standardized image obtainment methods may reduce this limitation. Overall Ning et al. found telemedicine interventions in otolaryngology to be beneficial and viable for screening, consultations, pre-operative assessments, and post-operative follow-up with high satisfaction among patients and providers [11]. A pilot study also found that telemedicine otolaryngology visits reduce costs for patients and peripheral healthcare systems [12]. As healthcare continues to evolve it is critical to consider telemedicine as it can lead to improved patient outcomes for individuals in rural and lower socioeconomic areas where access to specialty care is scarce.

A 2018 census stated that 37 million, or 35.3% of children in the U.S., have Medicaid [13]. The delay in critical treatment for this patient reflects the limited access to specialty care Medicaid patients face. While information on the differences in access to care between Medicaid patients and those with private insurance is scarce, evidence from comparison of adults with cancer shows that uninsured, medicare, and Medicaid patients have lower odds of receiving care at high-volume hospitals, which tend to have the most up-to-date facilities and access to specialist care [14]. Difficulty in connecting Medicaid patients with specialists is a known issue, with close to 60% of community health centers reporting difficulty with specialist referrals, and 49% citing administrative requirements as a barrier to providing adequate care [15]. This case provides a concrete example of how delays to treatment caused by administrative requirements can both negatively impact patient care and cause emotional and financial hardship to a patient’s family.

## Conclusions

This 14-year-old patient presented with a history of facial swelling and progressive left eye proptosis which was eventually diagnosed as JOF. JOF requires a careful correlation of clinical and radiologic factors for appropriate diagnosis and treatment. Due to insurance restrictions, long waits for appointments, access to specialized care in a semi-rural setting, travel distances, and the complexity of the patient’s case, treatment was delayed nearly two months from the initial presentation which could have led to significant long-term complications. This story is specific to our patients, but similar stories are experienced by numerous families and patients when seeking effective, appropriate, and equitable care to meet their needs. Patients with complex medical cases, lack of widespread local coverage, those who live in rural areas, and those who have lower socioeconomic backgrounds and language barriers may face similar added complexity in obtaining adequate care. It is critical for providers to recognize and anticipate these complicating factors to minimize their effect on patient outcomes. Improving access and payment for telemedicine, improving communication and coverage between payers and health systems/care providers, having mechanisms to fast track time-dependent cases such as these, avoiding unnecessary wait times, and receiving feedback from families and care providers can minimize added stress and negative patient outcomes.
